# Molecular assemblies built with the artificial protein Pizza

**DOI:** 10.1016/j.yjsbx.2020.100027

**Published:** 2020-05-28

**Authors:** Jeroen P.M. Vrancken, Jana Aupič, Christine Addy, Roman Jerala, Jeremy R.H. Tame, Arnout R.D. Voet

**Affiliations:** a Laboratory of Biomolecular Modelling and Design, Department of Chemistry, KU Leuven, Celestijnenlaan 200G, 3001 Leuven, Belgium; b Department of Synthetic Biology and Immunology, National Institute of Chemistry, Hajdrihova 19, 1000 Ljubljana, Slovenia; c Graduate School of Medical Life Science, Yokohama City University, 1-7-29 Suehiro, Yokohama, 230-0045 Kanagawa, Japan

**Keywords:** Protein design, Structural biology, Protein nanoparticles, Biotechnology

## Abstract

Recently an artificial protein named Pizza6 was reported, which possesses six identical tandem repeats and adopts a monomeric β-propeller fold with sixfold structural symmetry. Pizza2, a truncated form that consists of a double tandem repeat, self-assembles into a trimer reconstructing the same propeller architecture as Pizza6. The ability of pizza proteins to self-assemble to form complete propellers makes them interesting building blocks to engineer larger symmetrical protein complexes such as symmetric nanoparticles. Here we have explored the self-assembly of Pizza2 fused to homo-oligomerizing peptides. In total, we engineered five different fusion proteins, of which three appeared to assemble successfully into larger complexes. Further characterization of these proteins showed one monodisperse designer protein with a structure close to the intended design. This protein was further fused to eGFP to investigate functionalization of the nanoparticle. The fusion protein was stable and could be expressed in high yield, showing that Pizza-based nanoparticles may be further decorated with functional domains

## 1 Introduction

Natural protein cages have a broad range of biological functions, such as storing, protecting, and distributing small molecules or ions (Ueno et al., 2009). For many years, there has been a considerable interest in adapting these molecular scaffolds for biotechnology, and many such systems have been described. The most widely used cages are virus capsids and mammalian ferritins (Nassal et al., 2008, Han et al., 2014). Over the last three decades, synthetic biology has increasingly explored the design, re-engineering, and decoration of more or less novel protein cage systems, each with their own challenges (Seeman, 1982, Lai et al., 2012, Yeates, 2011). As with any protein design, a major difficulty is to prevent aggregation and misfolding. However, for high molecular weight complexes, artificial proteins may also show a lack of control over the assembly (Dill and MacCallum, 2012, Yeates et al., 2016). The most widely-used method to construct artificial protein assemblies is to combine domains that encode different symmetry elements into one protein subunit, so that the final structure is achieved by homo-oligomerization. The group of Yeates for example looked at different geometric architectures and how to achieve these by fusing two simple and prevalent symmetry elements, calling their method the “nanohedra design strategy” (Padilla et al., 2001). A similar approach was used by the group of Raman and colleagues, who connected into one peptide chain self-associating polypeptides and naturally occurring coiled-coils with different self-association symmetry. As a result of the different symmetry elements built into the peptide, it oligomerizes into sphere-like particles (Raman et al., 2006).

Besides nanohedral and sphere-like architectures, 2D crystalline assemblies can be created via these design strategies as well, as demonstated by Sinclair et al. (Sinclair et al., 2011). However, for both strategies the control over the relative orientation of the different domains within a monomer can still be challenging, not only due to the interactions between the protein subunits, but due to the linker as well. The composition and length of the linker connecting the different domains may strongly influence the internal orientation and flexibility (Sakai et al., 2014).

While the examples above rely on fusing peptide elements which assemble into symmetrical oligomers, one can also design a single monomer in which several unique elements pair into subdomain assemblies. This kind of polyhedral assembly, developed by the group of Jerala, is known as protein origami (Gradis̆ar et al., 2013). Given the added complexity of precisely orienting the subunits, as well as their folding, it is no surprise that significant effort has been required to develop computational methods explicitly for cage design, allowing more precise control over the final assemblies. Several large protein cages have now been reported, including a 60-subunit protein icosahedron from the group of Baker, which assembles purely through symmetrically designed protein interfaces (Hsia et al., 2016). In principle, the *ab initio* design of new proteins may lead to more versatile scaffolds, and in turn aid the development of molecules with a broader range of functions and applications. Nevertheless, the difficulties in the design of even simple globular proteins show that the re-design of natural building blocks provides a more secure route to unique and stable structures (Lapenta et al., 2018). For example, self-assembling cages (SAGEs) were created by the group of Woolfson using *de novo* designed peptide-based hubs that self-assemble into cage systems. Later, these particles were further redesigned into pSAGE, allowing them to display proteins that are both on and in the cage-system, leading to a variety of functions such as a nanoreactor (Fletcher et al., 2013, Ross et al., 2017).

Within bio-medicine and material science, self-assembling proteins are being developed with particular applications in mind. While bio-medicine focuses more on the decoration of protein nanoparticles or specific interactions with cellular effectors, material science generally focuses on creating hybrid constructs with inorganic nanoparticles, such as multi-dimensional arrays of nanodots assembled with very high precision. Examples in biomedicine include the development of vaccines (De Santis et al., 2017) and biosensors (Schuster, 2018), formation of cage-systems for encapsulation (Wörsdörfer et al., 2011), and tissue regeneration and engineering (Zhang et al., 2003), while in material science applications include memory storage (Prime et al., 2009), chemical sensing (Schatz et al., 2006), and protein nanowires (Rengaraj et al., 2017).

Here we present new building blocks consisting of two protein elements, one derived from Pizza proteins, while the other is an α-helical coiled-coil. Pizza proteins were *de novo* designed by our group as perfectly symmetric β-propellers (Voet et al., 2014). One repeat, or subunit, of a Pizza protein contains only 42 residues and is here referred to as a “blade”. The suffix following ‘Pizza’ indicates the number of blades in each monomer. Due to their sixfold symmetry, Pizza proteins have the tendency to assemble as highly stable, six-bladed structures. For the construct Pizza*n*, the number of subunits in the self-assembled oligomer is therefore the least common multiple of *n* and six, divided by *n*; e.g. Pizza4 forms a trimer while Pizza5 forms a hexamer. Pizza2, containing only two blades, has a very strong tendency to trimerize into a single six-bladed structure; other oligomeric forms are not found at detectable levels in solution. It therefore makes an excellent building block, encoding a threefold symmetry axis, for inclusion in novel protein nanoparticles. Pizza2 was redesigned into a metal binding protein (nvPizza2-S16H58) that also exhibits catalytic activity (Clarke et al., 2019). This protein not only has the ability to form a trimer, but in the presence of CdCl_2_ two trimers can dimerize, forming a protein sandwich filled with a nanocrystal of cadmium chloride, with precisely 19 atoms (Voet et al., 2015).

In this research we investigated novel building blocks consisting of metal-binding Pizza proteins together with hexameric or dimeric coiled-coils with a parallel or anti-parallel orientation. The ability of these fusion proteins to assemble according to the association preferences of each domain was examined and used to determine whether uniform and symmetric nanoparticles could be created from the two building blocks with minimal computational effort.

## 2 Materials and methods

### 2.1 Construction of plasmids

Linear DNA constructs encoding the fusion proteins were ordered from Integrated DNA Technologies (IDT, Integrated DNA Technologies) (Supplementary Table 1). These constructs consist of the base protein, Pizza2-S16H58, with different coiled-coils connected by sequence GSTGS or SGTGS. A silent BamHI restriction site was placed in the linker sequence to facilitate exchange of either domain separately, and can also be used to shorten the linker to identify the optimal linker length via custom made PCR primers (Supplementary Table 1). All the constructs utilized NdeI and XhoI restriction sites to allow the full-length PCR product to be inserted into pET28 (novagen).

### 2.2 Expression

pET28 carrying the inserted designer gene was transformed into *Escherichia coli* BL21 (DE3) cells using standard protocols. Cells were grown at 37°C to an optical density of about 0.6 at 600 nm (${\text{OD}}_{600}$) before expression was induced by adding β-D-1-thiogalactopyranoside (IPTG) to a final concentration of 0.5 mM, the cells were further cultured at 22°C for 20 h. Cultures were centrifuged and the pellet was resuspended in lysis buffer (200 mM NaCl, 50 mM NaH_2_PO_4_, and 10 mM imidazole pH 8.0).

### 2.3 Purification

Cells were lysed by sonication and centrifuged. The protein was purified from the supernatant using nickel-nitrilotriacetic acid (Ni–NTA). After loading, the resin was washed with buffer consisting of 200 mM NaCl, 50 mM NaH_2_PO_4_ pH 8.0 and 50 mM imidazole. The protein was eluted using the same buffer but with 600 mM imidazole.

After a first round of IMAC purification, fractions containing the protein (as indicated by SDS–PAGE) were collected and incubated with thrombin (Sigma–Aldrich BVBA) to remove the N-terminal his-tag. Following dialysis to remove the imidazole, IMAC purification was performed again to remove uncleaved protein and histidine rich impurities.

Next, proteins were further purified and characterized by Size Exclusion Chromatography (SEC, ÄKTA pure, GE Healthcare Life Sciences). Samples were loaded onto a HiLoad 16/600 Superdex 200 pg column equilibrated with 200 mM NaCl and 20 mM 4-(2-hydroxyethyl)-1-piperazineethanesulphonic acid (HEPES) pH 8.0 buffer. Fractions were analysed by both SDS–PAGE and Dynamic Light Scattering (DLS) to estimate the oligomerization state and dispersity. Elution times were compared to a standard curve, and oligomerization states were estimated from the theoretical molecular weight of the monomers. Only fractions that were both pure and monodisperse were combined and used for further protein characterization.

Monodisperse samples were also analyzed by analytical SEC. 200 μl of a 2 mg/ml sample was injected onto a Superdex 200 Increase 10/300 column equilibrated with 200 mM NaCl and 20 mM HEPES pH 8.0 to observe the elution profile.

### 2.4 Dynamic light scattering

DLS (ZetaSizer Nano ZS, Malvern) was used to analyze samples at 25°C for their monodispersity and to obtain an estimate of the radius of gyration (${\text{R}}_{g}$). The software used was provided by the manufacturer. The following parameters were used for the calculation: viscosity 0.891 cP, dielectric constant 79, and refractive index of 1.59, as only HEPES buffer systems were used (Mével et al., 2008).

### 2.5 Analytical ultra-centrifugation analysis

AUC was performed with an Optima XL-I analytical ultracentrifuge (Beckman-Coulter, Fullerton, California, USA) and an An-50 Ti rotor. Epon two-channel centerpiece cells with sapphire windows were used. For each experiment, 400 μl of the protein sample was used with 420 μl reference buffer, 200 mM NaCl and 20 mM HEPES pH 8.0. Two hours before each experiment, the rotor was equilibrated at 20°C in the vacuum chamber. Sedimentation-velocity experiments are performed at 40.000 rev min^−1^ and every ten minutes absorption scans at 280 nm were collected. Continuous-distribution c(s) analysis module in sedfit (Schuck et al., 2002) was used to analyse all the scans. Increments of 200 were used for the sedimentation coefficient, the frictional coefficient was allowed to float during the fitting. SEDNTERP was used to calculate proteins’ partial specific volumes, solvent density, and solvent viscosity (Lebowitz et al., 2002, Noguchi et al., 2019).

### 2.6 Electro-spray ionization mass spectrometry

Samples for Nanoflow Electro-Spray Ionization (ESI). were prepared by extensive dialysis against 20 mM ammonium acetate, and then adjusting the protein concentration to 10 μM by dilution with the same buffer. The mass spectra were obtained by Synapt G2 HDMS mass spectrometer (Waters) with a nanoESI source. The mass spectra were calibrated with (CsI) nCs + ions from m/z 1,000 to m/z 10,000. MassLynx version 4.1 software (Waters) was used for data processing and peak integration. The temperature of the ion source was set to 70°C. An aliquot of 3 ml of the sample solution was placed in a nanospray tip (HUMANIX, Japan) and electrosprayed at 0.8-1.0 kV.

### 2.7 Small-angle X-ray scattering (SAXS)

The P12 beamline at PETRA-III synchrotron (DESY, Hamburg, Germany) was used to measure the scattering curves (Blanchet et al., 2015). The incident wavelength was 1.24 Å, with a Pilatus 1 M detector positioned 2 m from the sample, allowing data collection for the scattering vector between 0.028 and 6.7 nm^−1^. A series of four concentrations ranging from 1 to 13 mg/ml was used for each protein to detect any potential concentration dependency effects. Data were collected over 20 frames lasting 0.05 s for each sample. Blank scans, of buffer without protein, were measured before and after each protein sample and were subtracted from sample scattering curves. A reference sample with known molecular weight, namely Bovine Serum Albumin (BSA), is also measured. Via the measured I_0_ of BSA and its molecular weight, the molecular weight of the measured protein samples can be calculated. Curves from a low and a high concentration sample were carefully merged to improve signal-to-noise ratio or to avoid concentration dependent effects. If a suffiction signal-to-noise ratio is present and inspection showed no concentration dependence and sufficient, curves obtained at high protein concentration were used for further analysis. Determination of model free parameters was carried out with PRIMUS (Konarev et al., 2003) and Scatter software (Förster et al., 2010). DAMMIF (Franke and Svergun, 2009) and DAMAVER (Volkov and Svergun, 2004) programs were used for the initial *ab initio* shape modelling. A refined low resolution bead model was generated with the *ab initio* modelling program DAMMIN (Franke and Svergun, 2009). Molecular models of designed protein cages were constructed via homology modelling using the previously reported crystal structures of the Pizza protein and coiled-coil domains as templates (Voet et al., 2014, O’Shea et al., 1991, Lu et al., 2012, Zhou et al., 2008, Thomson et al., 2014, Spencer and Hochbaum, 2016). Homology modelling was carried out with the MODELLER program package (Webb and Sali, 2016). To account for the flexibility of the linker region between the Pizza2 and the coiled-coil domain, an ensemble of structures was generated with individual domains at different relative orientations. Theoretical scattering curves were calculated for all generated models and compared to the experimental SAXS profiles using the FoXS program (Schneidman-Duhovny et al., 2016). In case, no good fits were obtained for a single model structure, multi-state modelling with MultiFoXS was performed (Schneidman-Duhovny et al., 2016). MultiFoXS provides a list of best matching ensemble structures and their fit-to-ratio score (χ value).

### 2.8 Molecular dynamics simulations

MD simulations were performed using GROMACS 4.6.7 (Bekker et al., 1993, Berendsen et al., 1995, Lindahl et al., 2001, Van Der Spoel et al., 2005, Hess et al., 2008) and the Amber99sb force field. Simulations were run on the Flemish Supercomputer Centre’s HPC. Complexes were modelled using the Molecular Operating Environment software (MOE, Version 2015, Chemical Computing Group Inc. Canada). Backbones from the symmetrically arranged Pizza2 (PDB: 3WW7 (Voet et al., 2014)) and coiled-coils (PDB: 2ZTA (O’Shea et al., 1991), 2LW9 (Lu et al., 2012), 1 K1F (Zhou et al., 2008), 4PN9 (Thomson et al., 2014), 5EON (Spencer and Hochbaum, 2016)) were structurally linked. The models were placed in a cubic periodic box expanded by 1 nm in each dimension compared to the largest dimension of the protein. Next, the box was solvated with TIP3P water and sodium chloride ions to neutralize the overall net charge at a concentration of 0.01 mM. Initial energy minimization was performed by steepest descent and conjugated gradient. Simulations performed as isothermal-isochoric (NVT) and isothermal-isobaric ensembles (NPT) at 298 K and 1 bar maintained with v-rescale temperature coupling and isotropic Parrinello-Rahman pressure coupling for the NPT ensemble. Simulations were run for 10 ns, with frames saved every 1 ns. Analysis and visualization was performed with GROMACS tools and PyMOL (Schrödinger, 2015).

## 3 Results

### 3.1 Design of Self-Assembling Building Blocks

Inspired by the two-part polyhedral (Raman et al., 2006) and nanohedra (Padilla et al., 2001) design strategies, we set out to combine our self-assembling symmetric designer protein with different coiled-coils to obtain symmetric and uniform nanoparticles. Each design incorporates two six-bladed Pizza structures, based on nvPizza2-S16H58, held together by α-helical coiled-coils. Different coiled-coil sequences were used that form either a dimer (D) or hexamer (H) with a parallel (P) or anti-parallel (A) orientation.

**Fig. 1:**
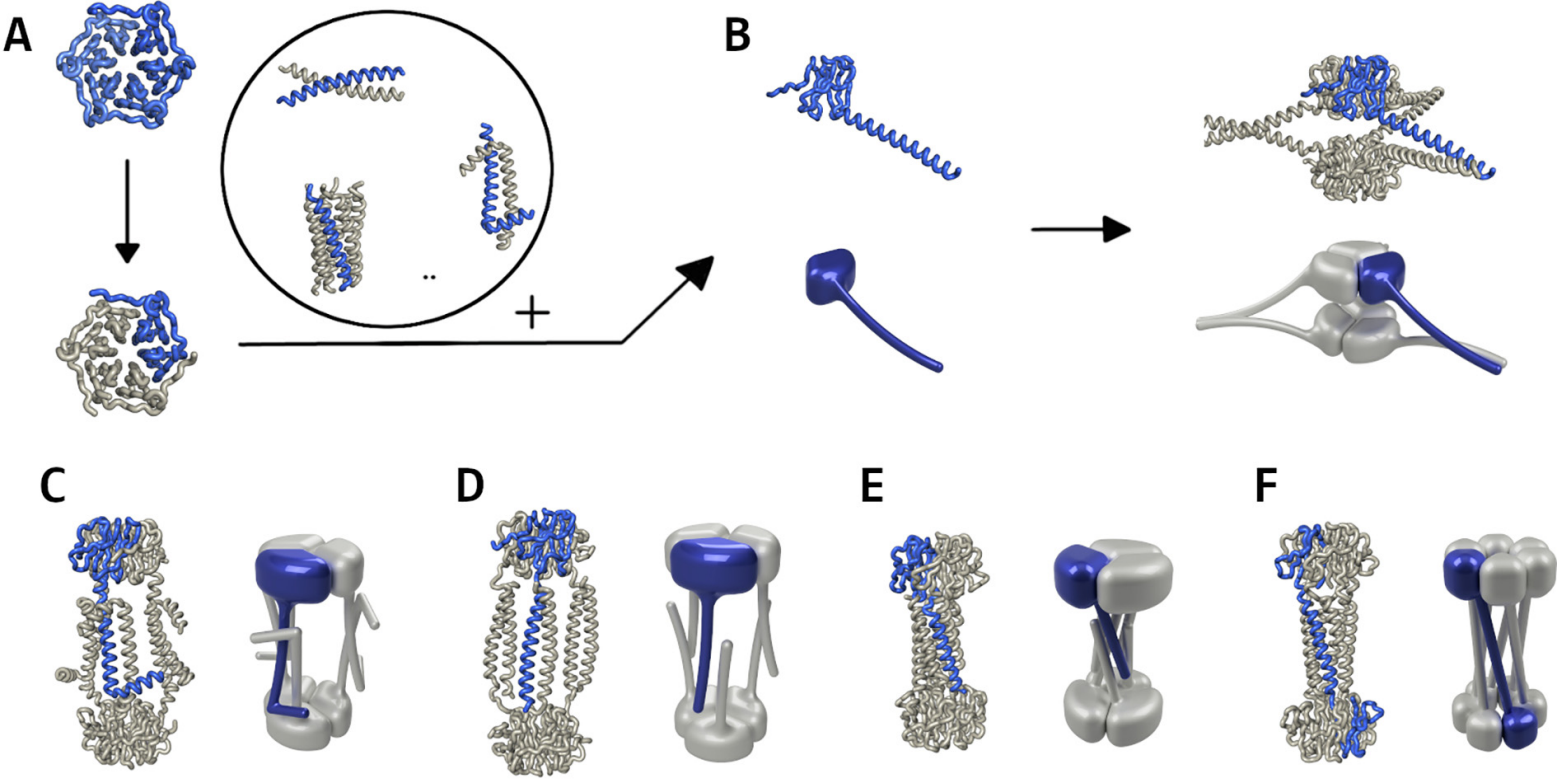
**Schematic flow of the fusion protein design strategy. A** depicts the truncation and permutation from Pizza6 to nvPizza2-S16H58. This mutant is combined with a coiled-coil, chosen from a small set of similar domains, to generate the fusion protein depicted in **B**. This fusion protein has the ability to self-assemble into a hexamer, using the dimeric coiled-coil and trimeric Pizza interfaces. Both the monomer and hexamer are shown as a cartoon representation and as a schematic figure. **C**-**F** depict the predicted oligomerization of other structures generated using other coiled-coil domains.

The position of the helices relative to the Pizza domain, and the connection between them, strongly influences the ability of each domain to assemble freely. Optimal positions to attach the coiled-coils to the Pizza protein were sought by examining the trimeric nvPizza2-S16H58 structure for non-conserved regions, which are likely to show higher flexibility. Linker sites were introduced by using circular permutations to move the preferred residues to the Pizza domain termini (Fig. 1D). These regions were predominantly found on the bottom face and sides of the Pizza protein, thus conserving the metal coordinating residues on the top for future applications.

This design method is not feasible with a parallel hexameric coiled-coil (Pizza-PH), as all six coiled-coil termini are aligned while the Pizza protein has a trimeric structure. In order to assume a similar assembly, this construct was designed with two single-bladed Pizza domains, one fused to each chain terminus of the coiled-coil domain.

As the length of the linker between the structured domains may influence their relative orientation, silent restriction sites were introduced allowing us to decrease the linker length one residue at a time. For each construct, versions with different linker lengths were tested for expression, and those with the highest yield of self-assembled complexes were retained. The optimum linker length for Pizza-PD and Pizza-AD were two and four amino acids respectively, while the other constructs showed the best results for linkers with five residues (Supplementary Fig. 1). The best-performing Pizza-PD, Pizza-AD, Pizza-nAD, Pizza-PH, and Pizza-AH constructs have theoretical molecular weights of 77.7 kDa, 87.6 kDa, 81.4 kDa, 77.3 kDa, and 74.8 kDa, respectively, for their hexameric assemblies.

### 3.2 Analysis of the oligomerization state

**Fig. 2:**
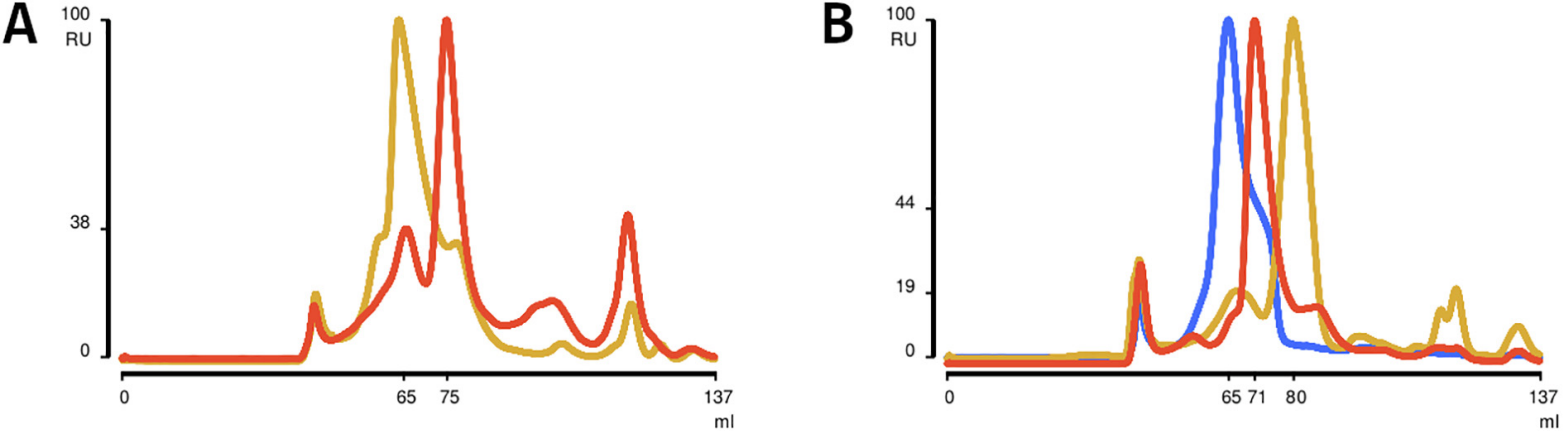
**Initial characterization of building blocks via SEC.** Chromatogram A shows the elution profiles of Pizza-AD in yellow, and Pizza-PD in orange. Pizza-PD shows one dominant peak, while Pizza-AD shows multiple smaller peaks. Chromatogram B shows the elution profiles of Pizza-PH in blue, Pizza-AH in yellow, and Pizza-nAD in orange. Both Pizza-AH and Pizza-nAD show one clear dominant peak, while Pizza-PH is susceptible to tailing.

After expression, all proteins were predominantly found in the soluble fraction. After purification by Immobilised Metal Ion Affinity Chromatography (IMAC) and removal of the N-terminal His_6_-tag, the proteins were purified by Size Exclusion Chromatography (SEC) resulting in elution profiles and a first estimate of the oligomerization states (Fig. 2).

The chromatograms of Pizza-PD, Pizza-nAD, and Pizza-AH all show one dominant peak corresponding to an estimated molecular weight of 81 kDa for Pizza-PD, 111 kDa for Pizza-nAD, and 56 kDa for Pizza-AH (See Table 1). Gel filtration is only an approximate method for estimating the molecular weight of roughly spherical proteins, and the results are compatible with Pizza-PD forming a hexamer. The result for Pizza-nAD may be due to a slightly higher oligomerization state than expected, or the highly extended shape. The lower than expected molecular weight for Pizza-AH indicates incomplete assembly. All fractions corresponding to these peaks were further analyzed for their monodispersity using DLS (Table 1 and Supplementary Fig. 2).

**Table 1:** **Analysis and comparison of proteins via different techniques.** The first three columns contain the sample and the theoretical weight of the monomer and hexamer. For SEC, and AUC the elution volume and sedimentation coefficient were converged to molecular weight. The DLS values are based on the main peak from SEC, except for Pizza-PH, which used the monodisperse fraction with the correct estimated molecular weight. AUC and ESI only show the values of the main peak.

Sample	Theo. Mw		SEC	DLS		AUC	ESI
	Mono. [kDa]	Hexa. [kDa]	Mw [kDa]	Z-Ave [d.nm]	PdI	Mw [kDa]	Mw [kDa]
Pizza-PD	12.9	77.7	81.8	8.24±0.02	0.048±0.007	75.0	77.7
Pizza-AD	14.6	87.6	194.9	11.92±0.1	0.109±0.006	/	/
Pizza-nAD	13.6	81.4	111.1	10.17±0.05	0.096±0.002	36.0	37.1
Pizza-PH	12.9	77.3	100.9	11.5±0.2	0.11±0.02	/	/
Pizza-AH	12.5	74.8	55.6	8.36±0.07	0.18±0.02	58.6	53.9
Pizza-PD-fluo	40.2	241.3	295.7	14.03±0.01	0.040±0.003	164.2	235

Several peaks are present in the elution profiles of Pizza-AD and Pizza-PH, strongly suggesting multiple oligomerization states. Both constructs show one large asymmetrical peak which broadens with a higher elution volume (‘tailing’). Tailing can be due to multiple conformations, partial unfolding, or degradation of the protein. Non-specific interactions with the column matrix are unlikely given the high-salt buffer used. Degradation was ruled out by SDS PAGE. The dominant peaks of Pizza-AD and Pizza-PH correspond to molecular weights of 195 kDa and 181 kDa, respectively, but protein was still found at much higher elution volumes in fractions that corresponded to the expected molecular weights of 68 kDa for Pizza-AD and 88 kDa for Pizza-PH. This fraction of Pizza-PH was analyzed using analytical SEC, but the new elution profile displayed a single tailing peak with an estimated weight closer to the former dominant peak of 181 kDa (Supplementary Fig. 3). Not only is the apparent size of the protein complex too high, the tailing suggests structural heterogeneity and an equilibrium between these complexes. Neither Pizza-AD nor Pizza-PH were characterized further.

### 3.3 Molecular weight estimation

**Fig. 3:**
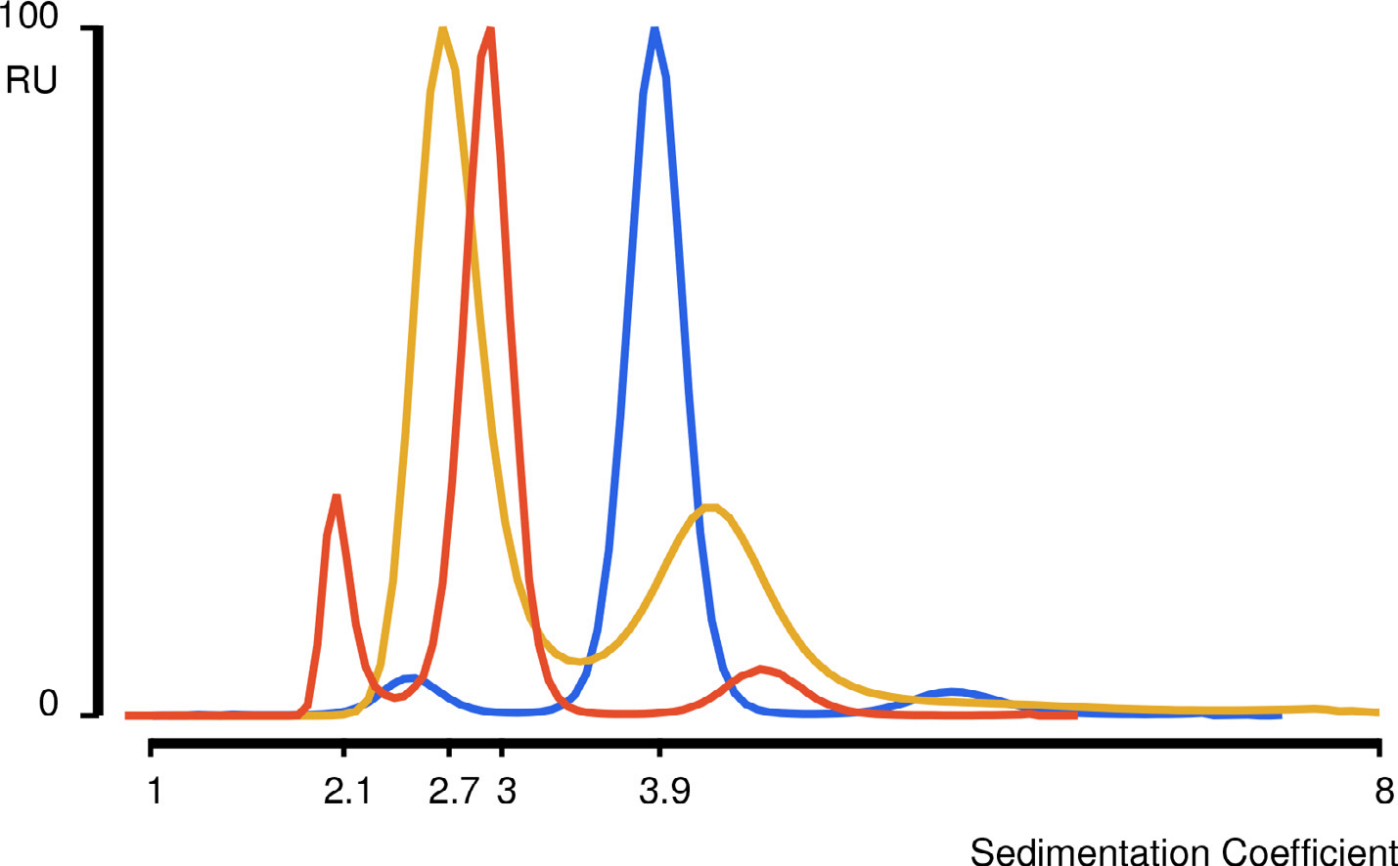
**Sedimentation profile via AUC.** Pizza-PD is shown in blue, Pizza-nAD in yellow, and Pizza-AH in orange. Only Pizza-PD shows a single peak, both Pizza-nAD and Pizza-AH show two peaks, suggesting multiple oligomerization states.

After SEC, the molecular weight was more accurately determined with analytical ultracentifugation (AUC) and electrospray ionization mass spectroscopy (ESI MS) (Fig. 3 and Supplementary Fig. 4). Sedimentation velocity (SV) analysis of Pizza-AH revealed two peaks with a sedimentation coefficient of 2.06 S and 2.94 S, with molecular weights of 58.6 kDa and 33.1 kDa (Table 1). Comparing intensity of the peaks, the smaller complex is roughly three times more abundant than the larger one. In addition, native MS indicates a molecular weight of 53.9 kDa, which is in agreement with the minor peak from the sedimentation profile. However, the theoretical weight of the hexamer is 74.8 kDa, indicating either incomplete assembly or degradation. The dominant peak from the sedimentation profile corresponds to the theoretical weight of the trimer.

Similar to Pizza-AH, the SV profile for Pizza-nAD also indicates two peaks with a molar ratio of 3:1. The dominant peak has a sedimentation coefficient of 2.66 S, corresponding to a molecular weight of 36.0 kDa, while the other peak has a sedimentation coefficient of 4.15 S and a molecular weight of 71.2 kDa, suggesting that the majority of the protein exists as a trimer. Furthermore, the native ESI MS data reveals a complex with a molecular weight of 37.1 kDa, confirming the trimeric state.

In contrast to Pizza-AH and Pizza-nAD, Pizza-PD appeared the most promising as it showed a clear sedimentation profile consisting of a single peak with a sedimentation coefficient of 3.87 S, and an estimated molecular weight of 75.0 kDa. Similarly, the ESI data indicates a molecular weight of 77.7 kDa, within 22 Daltons of the expected mass of the hexamer. The 5% over-estimate of mass from the SEC appears to arise from the shape of the complex.

### 3.4 In solution structure determination

**Fig. 4:**
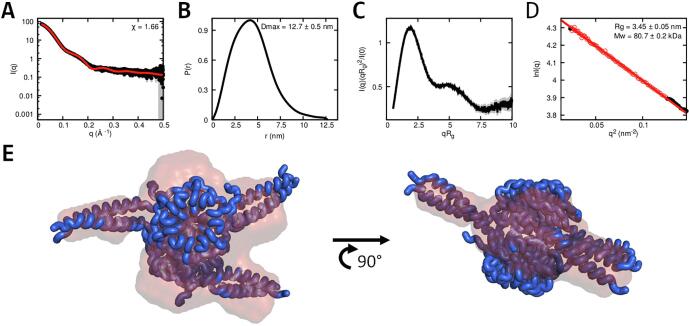
**Analysis of Pizza-PD via SAXS.** Graph **A** shows the experimental scattering curve in black and the scattering calculated from the models in red. The goodness-of-fit, χ, shown in the insets. Graph **B** shows the pair-distance distribution, P(r), derived from the scattering curve. The maximum distance between two atoms (Dmax) is 12.7±0.5 nm. The Kratky plot (**C**) displays a distinct second peak at higher scattering lengths. The Guinier plot (**D**), which shows the radius of gyration, Rg, is 3.45±0.05 nm, and molecular weight of 80.7±0.2 kDa. Panel **E** depicts the top and side view of the best scoring model (blue) aligned with the calculated DAMMIN envelope (orange).

Since Pizza-PD is uniform in solution, Small Angle X-ray Scattering (SAXS) was performed to obtain a low-resolution structure of the complex (Fig. 4). SAXS also provides the estimates of molecular weight and radius of gyration (Table 2). The molecular weight of Pizza-PD was determined to be 80.7±0.2 kDa, while the ${\text{R}}_{g}$ was 3.45 ± 0.05 nm.

**Table 2:** **SAXS analysis data and mass estimation.** For Pizza-PD, data collected using samples with high and low protein concentration were similar enough to allow merging to improve the signal-to-noise.

Sample	${\text{I}}_{0}$ [cm^−1^]	Ratio of fit [χ]	${\text{D}}_{\max}$ [nm]	${\text{R}}_{g}$ [nm]	Mw [kDa]	Oligomeric state
Pizza-PD	16830±48	1.66	12.7±0.5	3.45±0.05	80.7±0.2	6

The pair-distance distribution function, P(r), for Pizza-PD displays an asymmetrical peak with residual values at a higher size and ${\text{D}}_{\max}$ of 12.7 ± 0.5 nm in agreement with the estimated ${\text{D}}_{\max}$ from the initial design (Fig. 1). The Kratky plot also indicates a well-folded multi-domain structure, which corresponds to the three protruding coiled-coil pairs attached to the β-propeller.

Multiple models were generated from the design, and scored against the experimental scattering curve with FoXS (Schneidman-Duhovny et al., 2016). Different low-resolution models were generated and scored by DAMMIF and DAMMIN (Franke and Svergun, 2009). Comparison and scoring of these models occured via DAMAVER and SUPCOMB and were also manually observed via PyMOL (Volkov and Svergun, 2004, Kozin and Svergun, 2001, Schrödinger, 2015). The majority of these models were fairly similar and a representative model was aligned with the best scoring structure (Fig. 4). The envelope clearly shows three protruding elements from the main body, which fit the three coiled-coil pairs from the best scoring models.

**Fig. 5:**
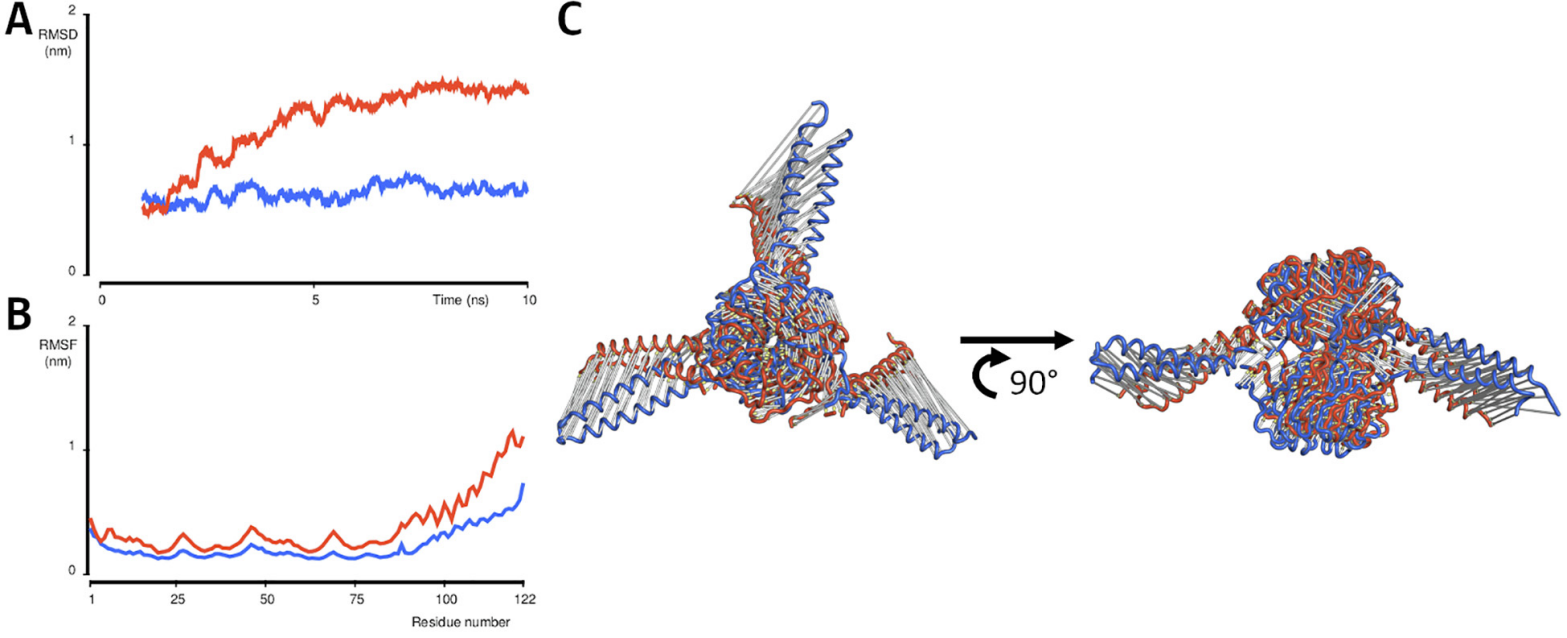
**Analysis of Pizza-PD via MD simulations.** Graph **A** and **B** display the RMSD and RMSF plots for both the designed model (orange) and the SAXS output (blue) over a simulation of 10 ns. The designed model shows more fluctuation than the SAXS output, but both models indicate more flexibility for the helices, which start at residue number 90. Panel **C** shows overlays of the starting model (blue) and final model (orange), both top and side view. During the simulation, the helices pivot around their connection to the Pizza domains, generating a model close to the SAXS output.

The main difference between the original design and the best scoring model is the orientation of the coiled-coils. The short flexible linkers connecting the Pizza and coiled-coil domains together allow some relative motion between them, hence short molecular dynamics (MD) simulations were run for 10 ns to confirm this motion for both the designed Pizza-PD model and the best fitting model from the SAXS experiments (Fig. 5).

Analysis of both trajectories revealed the individual domains are highly stable and confirmed the high mobility of the coiled-coils arises from the linkers. Interestingly, the symmetric starting model quickly adopted asymmetric orientations with the coiled-coils approaching the edges of the β-propellers. The final structures of the MD simulations therefore corresponded better to the SAXS profile than the design, confirming the high structural mobility.

### 3.5 Functionalization

To determine whether Pizza-PD could act as a scaffold to carry additional protein domains, an enhanced Green Fluorescent Protein (eGFP) was added to the C-terminus, resulting in a three domain construct (Pizza2 unit, coiled-coil segment, and eGFP). This new protein, Pizza-PD-fluo, was expressed and purified in the same way as the parent protein. After purification, the SEC elution profile displayed multiple peaks (Supplementary Fig. 5), possibly due to the self-interaction of the eGFP domains, resulting in conformational heterogenity. The dominant peak was first checked for monodispersity using DLS (Supplementary Fig. 2) before being subjected to AUC and ESI. The sedimentation profile indicated several forms of the protein, but mass spectrometry confirmed the intended hexameric complex was present in solution. SAXS was carried out to provide an independent assessment of the protein size and shape.

**Fig. 6:**
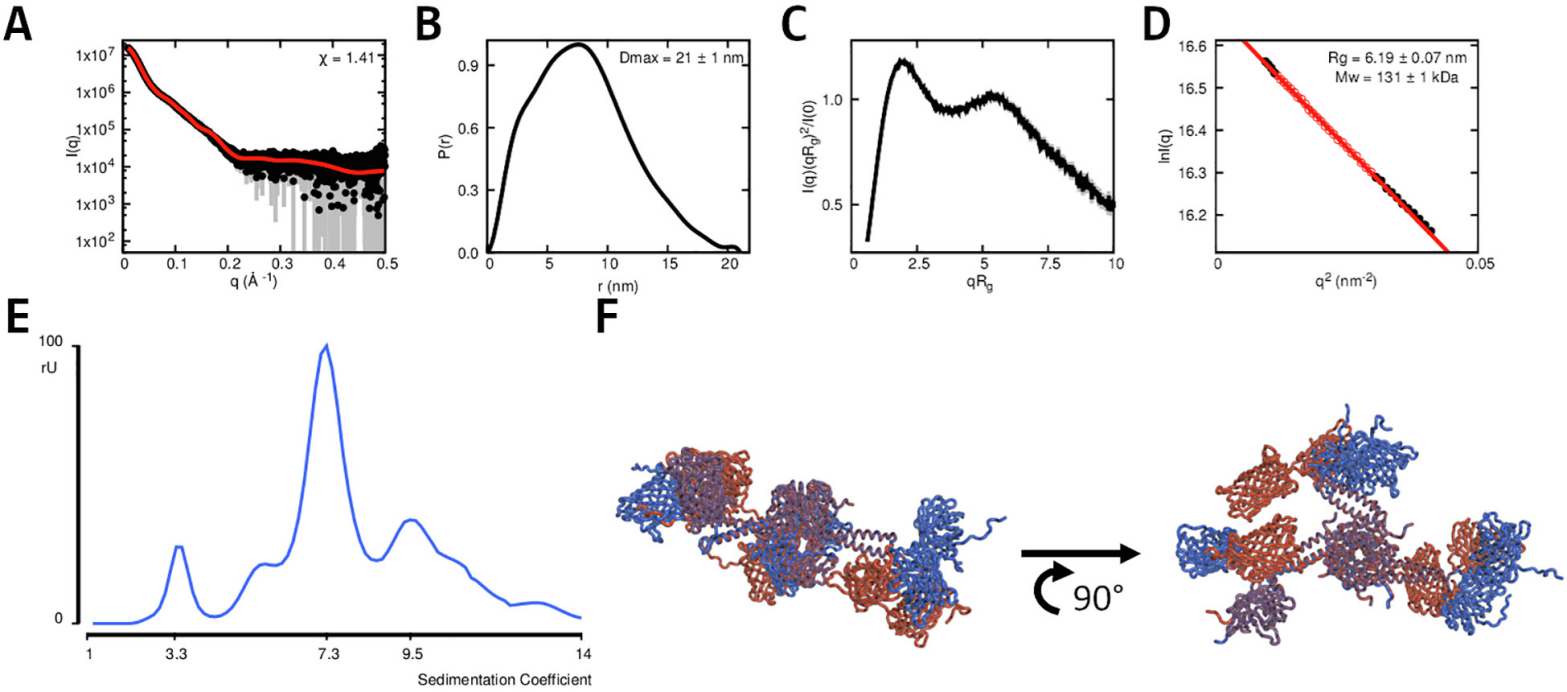
**Characterization of Pizza-PD-fluo via SAXS and AUC. A**-**D** shows the SAXS results for Pizza-PD-fluo. Graph **A** shows the experimental scattering curve in black and the scattering calculated from the ensemble in red. The goodness-of-fit, χ, shown in the insets. Graph **B** displays the pair-distance distribution functions, P(r), as obtained by the SAXS experiment and the obtained ${\text{D}}_{\max}$ of 21±1 nm. The Kratky plot (graph **C**) displays two distinct peaks and the Guinier plot (graph **D**) shows the radius of gyration, Rg, of 6.19±0.07 nm and a molecular weight of 131±1 kDa. Graph **E** shows the sedimentation profile of the purified protein and the ensemble of best scoring SAXS models can be seen in panel **F**.

Compared with Pizza-PD, the pair-distance distribution for Pizza-PD-fluo indicates a bigger structure, with a ${\text{D}}_{\max}$ of 21 ± 1 nm, and the Kratky plot clearly hints that the complex contains multiple well-folded domains. To examine the relative positions of the six added eGFP domains, new structural models were built from the best scoring Pizza-PD model. However, no single model could be obtained with a satisfactory fit for the SAXS data, which is in accordance with the conformational heterogenity observed with SEC profiles. MultiFoXS was then used to obtain an ensemble of structures that could explain the observed scattering. The best match was given by an ensemble of two structures that mainly differed in the orientation of the eGFP units (Fig. 6**F**).

## 4 Discussion

Inspired by the modular approach to design symmetric and uniform nanoparticles, we explored the possibility of utilizing the symmetric Pizza protein as a modular building block. Coiled-coils with different symmetry elements were attached to a trimeric form of Pizza to create five different designs. Each of these proteins could be expressed and purified, but two proteins, namely Pizza-AD and Pizza-PH. These two constructs appeared to form assemblies with a molecular weight far above the expected mass. Pizza-AD was based on the short anti-parallel dimerization coiled-coil found in class X myosin (Lu et al., 2012). Structural analysis of the myosin X motor have described the dimerization domain and its effects in detail (Umeki et al., 2011, Ropars et al., 2016). Umeki et al. demonstrated that dimerization is induced by the binding of phosphatidylinositol-3,4,5-triphosphate to the pleckstrin homology domain following the anti-parallel coiled-coil. Although dimerization still occurs to some extent without the binding of phospholids (Umeki et al., 2011). Ropars et al. showed that the motility of the myosin is dependent on the flexibility of the lever arm containing the dimerization region (Ropars et al., 2016). This coiled-coil was chosen as a dimerization domain since the presence of three copies in the design was believed sufficient to create a single, stable complex. In practice however, higher oligomerization states were obtained presumably due to flexibility present in the coiled-coil or the linker in combination with somewhat protruding coiled-coils, as seen from Pizza’s symmetry axis.

To date, no form of Pizza has been demonstrated in which six separate polypeptides combine into a single sixfold symmetric β-propeller. Pizza-PH was designed in an attempt to use a sixfold coiled-coil to bring six Pizza1 repeats in close proximity by the means of a sixfold symmetric parallel oriented α-helical barrel. As this strategy did not succeed in guiding the assembly, it appears that a minimum of two repeats is required to form a folding nucleus from which the final β-propeller form may emerge.

The other designed constructs could assemble, but not always as intended. Pizza-nAD was predominantly a trimer, implying that only its Pizza2 units were able to assemble. We conjecture that the fast oligomerization of Pizza2 limits the general freedom of the associated coiled-coils, resulting in a deadlocked plateau between the trimer and the hexamer (Deeds et al., 2012). As their freedom is limited, coiled-coil domains attached to the same Pizza propeller may prefer to associate, rather than linking separate propellers, reducing their ability to form the desired hexamer. Subsequently, given the presence of incomplete assemblies, one coiled-coil can protrude from the incomplete assembly and interact with a similar incomplete assembly, leading to higher oligomerization states, as seen for Pizza-AH.

Pizza-PD is the only protein design that gave a well-expressed, stable, monodisperse, and correctly assembled protein nanoparticle. This is presumably due to its different shape compared to the other more prolate assemblies. Pizza-PD’s coiled-coil pairs lie perpendicular to the symmetry axis of the Pizza propeller, making alignment with the other helices unnecessary to achieve the final assembly. Our results reflect to some extent those of the Arai group, whose nanohedra design strategy showed bi-modular building blocks that can adopt several oligomerization states, not only the intended one (Kobayashi et al., 2015).

These results demonstrate the requirement for computational optimization to improve the design success. Furthermore, they also demonstrate the use of Pizza protein as a building block that can assemble into nanoparticles and other oligomeric forms. The limiting factor described here appears to be the flexibility of the linker between both protein units and the stability of the coiled-coils fragments. Although specificity is unlikely to be an issue for the purified protein constructs, the coiled-coils may be only transiently folded if they protrude from the main body of the complex and make almost no non-covalent contact with it or initiate interactions with similar folded assemblies. Symmetry may be broken to achieve a lower-energy asymmetrical state by balancing local and global interactions, additionally this can also lead to different oligomerization states and a deadlocked plateau (Grigoryan and DeGrado, 2011, Lupas and Bassler, 2017, Deeds et al., 2012). One solution may include designer-helices to ensure optimal affinity of the helices and ensure correct stoichiometry (Fletcher et al., 2017). Nevertheless, even with designer proteins there might be issues with specificity, so unwanted oligomerization states should be discouraged by the “negative design” strategy (Fletcher et al., 2012). Another approach worth exploring is the replacement of the coiled-coils with other designer protein building blocks such as derivatives of the Tako and Ika proteins, which have eightfold or fourfold symmetry (Noguchi et al., 2019). These more rigid domains may allow more complex shapes to be created in combination with Pizza. Alternatively, it may be possible to functionalize the scaffold itself, avoiding the need for accessory protein domains to create novel materials. Assemblies of Pizza have already been demonstrated that rely on metal ions to hold copies of the protein together (Voet et al., 2015). Here we have shown that Pizza-based proteins can assemble into nanoparticles which in the future may serve as a framework for a variety of applications such as antigen presentation for enhanced vaccine design (Marcandalli et al., 2019), scaffolding enzymes to create a catalytic cascade (Wang et al., 2019), or as larger self-assembling scaffolds. As there is currently much attention focused on the development of cost effective, artificial matrices that can support cell and tissue growth, an attractive route to such materials can be the decoration of supramolecular Pizza complexes with suitable cell-binding domains. In addition, when observing the opposing orientation of the Pizza units in Pizza-PD, it may be feasible to expanded the assembly into a larger double-layered systems, as earlier seen by Kepiro et al. whose assemblies showed antimicrobial activity (Kepiro et al., 2020). In such case, two Pizza proteins may be brought face-to-face by generating a heteromeric or homomeric fusion protein, resulting in additional functionality as respectively one or both sides of layers can be functionalized, prior to their assembly into layers. However, we only investigated Pizza complexes with homomeric coiled-coils with limited computational design.

To conclude, we have successfully designed multiple stable and monodisperse building blocks that self-assemble into symmetric and uniform nanoparticles. From these designs Pizza-PD fits the experimental results and thus was used to functionalize with eGFP, resulting in Pizza-PD-fluo. This protein was expressed and purified similarly to its parent, Pizza-PD. Pizza-PD-fluo led to monodisperse samples that confirmed the design, thus confirming the possibility of utilising the proteins as scaffolds.

## Author’s contributions

JPMV performed and analyzed all the experiments. JA performed SAXS experiments. CA performed AUC experiments. ARDV, JRHT, and RJ conceived and supervised all experiments. All authors contributed to the writing of the manuscript.

## Declaration of Competing Interest

The authors declare that they have no known competing financial interests or personal relationships that could have appeared to influence the work reported in this paper.
