# Supplementary material for: Molecular assemblies built with the artificial protein Pizza
Source: J Struct Biol X. 2020 May 28;4:100027. doi: 10.1016/j.yjsbx.2020.100027 (PMC7337062; doi:10.1016/j.yjsbx.2020.100027)
Supplement: Supplementary data 1 [file mmc1.pdf]

Supplementary material to

The Pizza designer protein as a base for small cage-like structures

Jeroen PM Vrancken[a], Jana Aupič[b], Christine Addy[c], Jerala Roman[b], Jeremy RH Tame[c] and Arnout RD Voet*[a]

[a]*Laboratory of Biomolecular Modelling and Design, Department of Chemistry, KU Leuven, Celestijnenlaan 200G, 3001 Leuven, Belgium*
[b]*Department of Synthetic Biology and Immunology, National Institute of Chemistry, Hajdrihova 19, 1000 Ljubljana, Slovenia*
[c]*Graduate School of Medical Life Science, Yokohama City University, 1-7-29 Suehiro, Yokohama, 230-0045 Kanagawa, Japan*

Supplementary Table 1
DNA sequences of re-designed constructs.

Protein		DNA sequence
Pizza-PD	1	CATATGAGCA ATACCCAGAC GGTCTTACCG TTCACTGGCT TAAACACCCC ATCGGGTGTG GCCGTTGATT
	71	CTGCCGGTAC CGTGTACGTG ACCGATCATG GCAACAACCG TGTCGTTAAG CTGGCCGCGG GTTCTAACAC
	141	CCAAACCGTG TTGCCATTCA CAGGATTAAA CACGCCAAAT GGCGTCGCCG TTGACAGCGC GGGTACTGTA
	211	TATGTTACCG ATCATGGTAA CAATCGCGTC GTAAAACTGG CAGCAGGATC CGGCACTGGT AGCCGCATGA
	281	AGCAGCTGGA GGATAAAGTC GAAGAACTGT TGTCAAAAAA CTATCATCTG GAAAACGAAG TTGCCCGTCT
	351	GAAGAAACTG GTCGGGGAAC GGTAAAAGCT TTAATAACTC GAG
Pizza-AD	1	CATATGAGCA ATACACAGAC CGTGCTGCCA TTTACTGGCC TTAATACCCC GAGCGGTGTA GCGGTAGATT
	71	CCGCCGGCAC CGTATACGTT ACTGATCATG GGAATAACCG TGTGGTTAAA TTGGCTGCTG GAAGCAATAC
	141	TCAAACCGTT TTGCCGTTTA CTGGCTTGAA CACTCCCAAT GGTGTGGCAG TCGATTCGGC CGGGACGGTC
	211	TATGTGACCG ATCATGGCAA CAACCGTGTG GTAAAACTGG CAGCCGGATC CGGCACCGGC AGTGAAAATA
	281	AGCAGGTGGA GGAGATCCTG CGCCTGGAAA AGGAAATCGA GGACCTGCAA CGCATGAAGG AACAGCAAGA
	351	ACTGTCGTTG ACAGAGGCAA GCCTGCAGAA GTTGCAAGAA CGCCGCGACC AAGAATAAAA GCTTTAATAA
	421	CTCGAG
Pizza-nAD	1	CATATGAGCA ATACACAGAC CGTGCTGCCA TTTACTGGCC TTAATACCCC GAGCGGTGTA GCGGTAGATT
	71	CCGCCGGCAC CGTATACGTT ACTGATCATG GGAATAACCG TGTGGTTAAA TTGGCTGCTG GAAGCAATAC
	141	TCAAACCGTT TTGCCGTTTA CTGGCTTGAA CACTCCCAAT GGTGTGGCAG TCGATTCGGC CGGGACGGTC
	211	TATGTGACCG ATCATGGCAA CAACCGTGTG GTAAAACTGG CAGCCGGATC CGGCACCGGC AGTGATATCG
	281	AGCAAGAATT GGAAAGGGCC AAAGCCTCGA TTCGGCGTCT GGAGCAGGAG GTCAACCAGG AGCGTAGCCG
	351	GATGGCGTAT TTACAGACTC TCTTAGCAAA ATAAAAGCTT TAATAACTCG AGCGGTAA
Pizza-AH	1	GAATTCCATA TGGCGGGCAC TGTTTACGTG ACGGATCACG GGAATAACCG CGTTGTGAAA CTGGCAGCCG
	71	GTTCCAATAC CCAGACCGTA CTGCCCTTTA CAGGTTTAAA CACCCCGAAT GGAGTTGCGG TAGACTCTGC
	141	CGGGACTGTC TATGTTACCG ATCATGGAAA TAACCGTGTG GTCAAATTGG CTGCCGGCAG TAATACACAG
	211	ACAGTACTCC CATTTACGGG TCTGAATACG CCTAACGGCG TCGCTGTCGA CAGCGGTTCA ACTGGCTCGG
	281	AACTTAAGGC TATTGCACAA GAATTTAAAG CAATCGCCAA GGAGTTCAAA GCGATTGCAT GGGAGTTCAA
	351	AGCGATAGCT CAGTAAAAGC TTTAATAACT CGAGCGGTAA
Pizza-PH	1	GAATTCCATA TGGCGGGCAC TGTTTACGTC ACCGACCACG GGAATAACCG TGTCGTGAAG CTGGCGGCAG
	71	GGTCCAATAC GCAGACGGTT TTGCCGTTCA CCGGCCTCAA CACCCCCAAT GGGGTCGCGG TGGATTCTGG
	141	ATCCACAGGC AGCGGAGAGA TTGCAAAATC ACTGAAAGAA ATAGCTAAGA GCTTAAAAGA AATTGCGTGG
	211	TCATTAAAAG AAATCGCCAA ATCTCTGAAG GGGTCGACCG GAAGTGCTGG TACAGTTTAC GTTACAGATC
	281	ATGGCAATAA CCGCGTTGTG AAACTAGCAG CTGGCAGCAA TACTCAAACT GTGCTGCCAT TTACGGGTCT
	351	TAATACTCCT AACGGTGTAG CCGTAGATTC GTAAAAGCTT TAATAACTCG AGCGGTAA
Pizza-PD-fluo	1	CATATGAGCA ATACCCAGAC GGTCTTACCG TTCACTGGCT TAAACACCCC ATCGGGTGTG GCCGTTGATT
	71	CTGCCGGTAC CGTGTACGTG ACCGATCATG GCAACAACCG TGTCGTTAAG CTGGCCGCGG GTTCTAACAC
	141	CCAAACCGTG TTGCCATTCA CAGGATTAAA CACGCCAAAT GGCGTCGCCG TTGACAGCGC GGGTACTGTA
	211	TATGTTACCG ATCATGGTAA CAATCGCGTC GTAAAACTGG CAGCAGGATC CCGCATGAAG CAGCTGGAGG
	281	ATAAAGTCGA AGAACTGTTG TCAAAAAACT ATCATCTGGA AAACGAAGTT GCCCGTCTGA AGAAACTGGT
	351	CGGGGAACGG GGCAGCGGCA GCATGGTGAG CAAGGGCGAG GAGCTGTTCA CCGGGGTGGT GCCCATCCTG
	421	GTCGAGCTGG ACGGCGACGT AAACGGCCAC AAGTTCAGCG TGTCCGGCGA GGGCGAGGGC GATGCCACCT
	491	ACGGCAAGCT GACCCTGAAG TTCATCTGCA CCACCGGCAA GCTGCCCGTG CCCTGGCCCA CCCTCGTGAC
	561	CACCCTGACC TACGGCGTGC AGTGCTTCAG CCGCTACCCC GACCACATGA AGCAGCACGA CTTCTTCAAG
	631	TCCGCCATGC CCGAAGGCTA CGTCCAGGAG CGCACCATCT TCTTCAAGGA CGACGGCAAC TACAAGACCC
	701	GCGCCGAGGT GAAGTTCGAG GGCGACACCC TGGTGAACCG CATCGAGCTG AAGGGCATCG ACTTCAAGGA
	771	GGACGGCAAC ATCCTGGGGC ACAAGCTGGA GTACAACTAC AACAGCCACA ACGTCTATAT CATGGCCGAC
	841	AAGCAGAAGA ACGGCATCAA GGTGAACTTC AAGATCCGCC ACAACATCGA GGACGGCAGC GTGCAGCTCG
	911	CCGACCACTA CCAGCAGAAC ACCCCCATCG GCGACGGCCC CGTGCTGCTG CCCGACAACC ACTACCTGAG
	981	CACCCAGTCC AAGCTGAGCA AAGACCCCAA CGAGAAGCGC GATCACATGG TCCTGCTGGA GTTCGTGACC
	1051	GCCGCCGGGA TCACTCTCGG CATGGACGAG CTGTACAAGT AAAAGCTTTA ATAACTCGAG CGGTAA

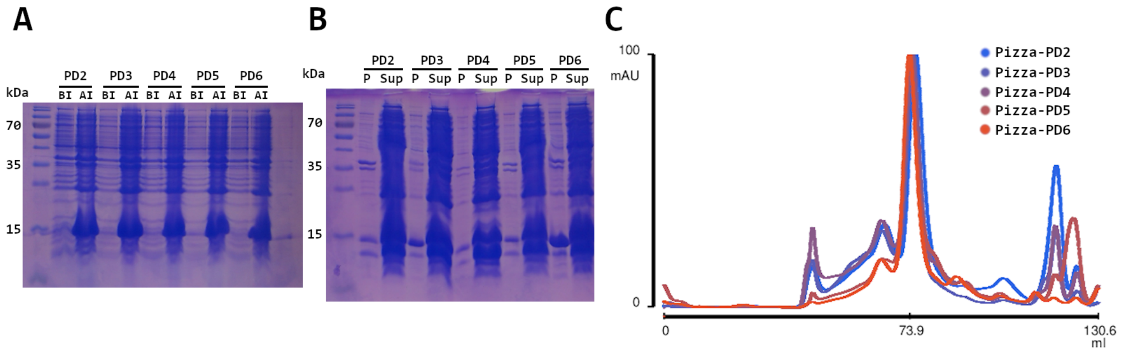

Supplementary Figure 1: Selection of optimal linker for Pizza-PD. Panel **A** shows before (BI) and after induction (AI) of Pizza-PD constructs with linkers differing in length. After lysis and centrifugation, samples from the pellet (P) and supernatant (Sup) were loaded on a separate gel (**B**). These gels indicate a good expression for all constructs, but slightly higher for linker length two and three. Constructs with a linker length of two and four also seem to have a smaller fraction of inclusion bodies. All constructs have a similar SEC elution profile (**C**). Finally, linker length of two was chosen due to these qualities and to limit the flexibility of the linker.

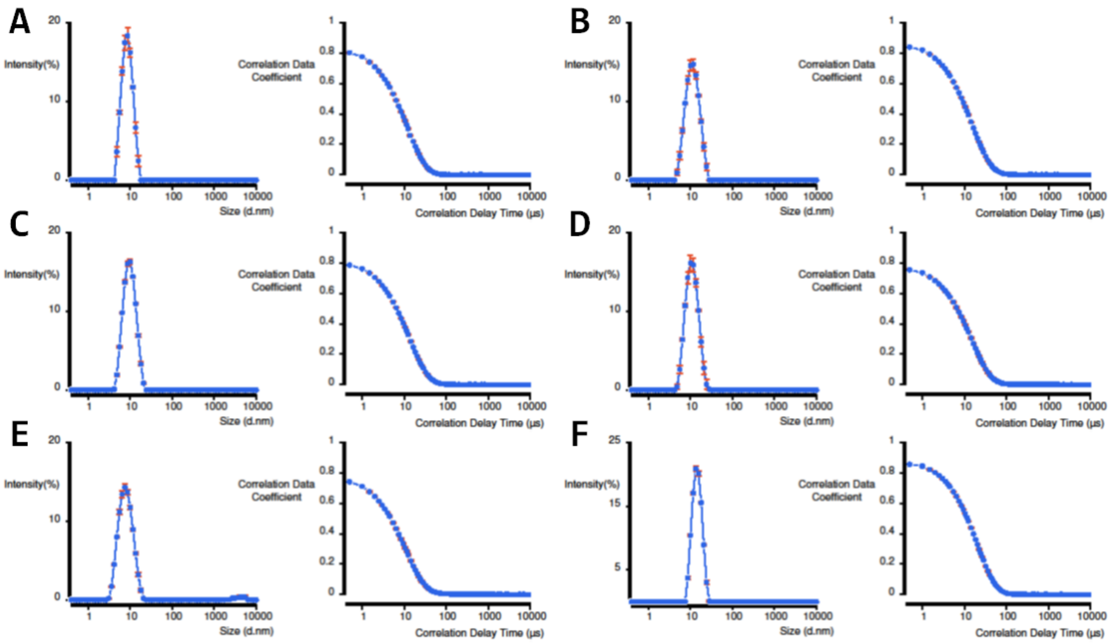

Supplementary Figure 2: DLS profiles of the main SEC fractions. A - F show the distribution profile and the correlation function of Pizza-PD, Pizza-AD, Pizza-nAD, Pizza-PH, Pizza-AH, and Pizza-PD-fluo, respectively. For Pizza-PH this fraction was collected and used for the analytical SEC (Supplementary Figure 3).

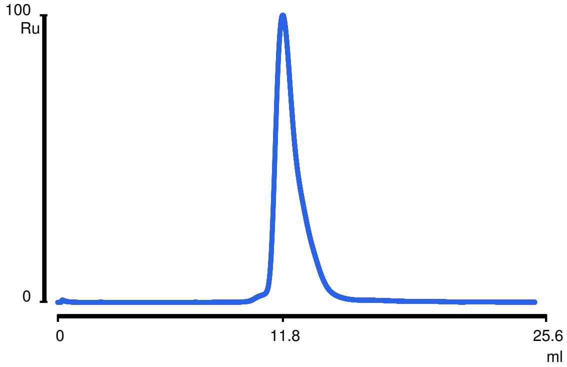

Supplementary Figure 3: Characterization of Pizza-PH via Analytical SEC. A monodisperse fraction located in the shoulder of the main peak (Figure 2) and corresponding with the expected theoretical molecular weight was injected on an analytical SEC column. This chromatogram shows a clear defined peak which is susceptible to tailing.

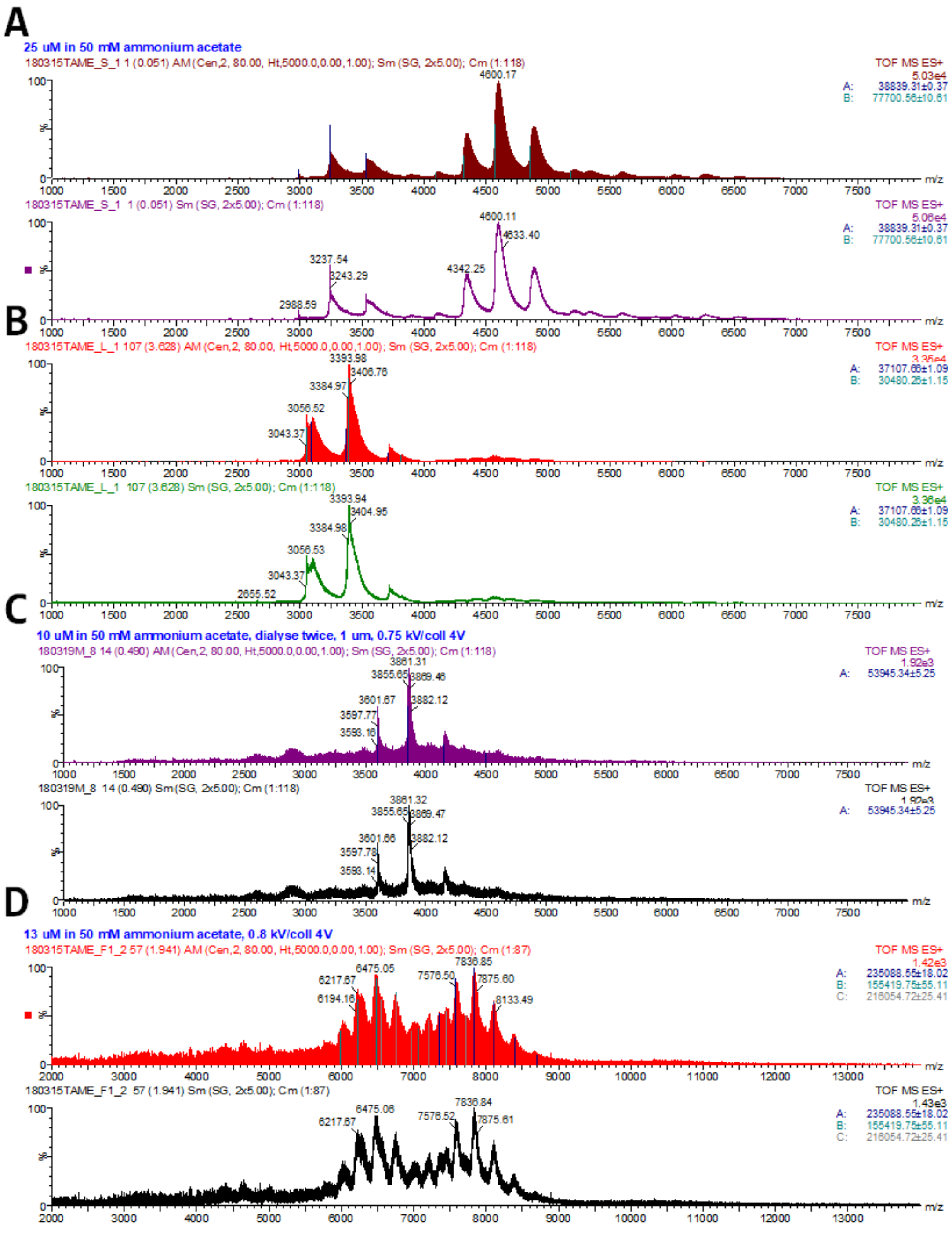

Supplementary Figure 4: Characterization of building blocks with ESI. A - D show the chromatograms of Pizza-PD, Pizza-nAD, Pizza-AH, and Pizza-PD-fluo, respectively.

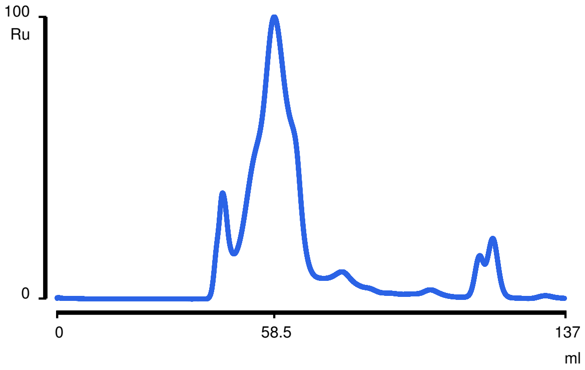

Supplementary Figure 5: Characterization of Pizza-PD-fluo via SEC. The elution profile of Pizza-PD-fluo shows one dominant peak with two shoulders.
